# The DNA methylation-regulated MCTP1 activates the drug-resistance of esophageal cancer cells

**DOI:** 10.18632/aging.104173

**Published:** 2021-02-11

**Authors:** Lingsuo Kong, Wan Yang, Lanren Chen, Liting Qian

**Affiliations:** 1 Department of Anesthesiology, West District of The First Affiliated Hospital of USTC, Division of life Sciences and Medicine, University of Science and Technology of China, Hefei 230031, Anhui, P.R. China; 2 Department of Radiotherapy, The First Affiliated Hospital of USTC, Division of Life Sciences and Medicine, University of Science and Technology of China, Hefei 3230031, Anhui, P.R. China

**Keywords:** esophageal cancer, drug-resistance, hypermethylation, MCTP1

## Abstract

Accumulating studies have demonstrated that drug-resistance remains a great obstacle for the effective treatment of cancers. Esophageal cancer is still one of the most common cancers worldwide, which also suffers from the drug-resistance during clinical treatment. Here we performed drug-resistance profiling assays and identified several drug-resistant and drug-sensitive esophageal cancer cell lines. The following methylation sequencing showed that the MCTP1 gene is hypermethylated in the drug-resistant esophageal cancer cells. As a result, the expression of MCTP1 is down-regulated in the drug-resistant esophageal cancer cells. Down-regulation of MCTP1 also affects the migration and apoptosis of esophageal cancer cells, as revealed by the wound-healing and apoptosis assays. Further investigations proposed two signaling pathways that might involve in the MCTP1-mediated drug-resistance of esophageal cancer cells. All these results suggested that MCTP1 activates the drug-resistance of esophageal cancer cells, which has implications for further design of new biomarker of esophageal cancer treatment.

## INTRODUCTION

Esophageal cancer (EC) is one of the most fatal malignancies worldwide, with an increasing incidence in the past few decades [1]. Extensive studies have made great progress on the treatment of EC patients [2, 3]. Due to the lack of early clinical symptoms, EC is often diagnosed at its advanced stages. Thus, the prognosis of EC patients remains poor with the overall 5-year survival rate less than ~20% [4]. In an attempt to improve the outcome of patients after surgery, EC patients are often treated with chemoradiotherapy to decrease tumor size. However, the chemoradiotherapy may enhance toxicity levels and possibly cause the resistance of the EC cells against the drugs [5, 6]. Thus, it is urgently needed to screen and identify new precise biomarkers that could predict the EC patients who may or may not respond well to the chemotherapy [7]. Thus, identifying new biomarkers is also useful to predict the treatment response of patients while improving their survival rates. To achieve this goal, we need to investigate the underlying mechanism that governs the chemoresistance of EC cells.

DNA methylation is the best-characterized epigenetic mechanism. The hypermethylated state of the promoter and enhancer regions tightly correlates with the transcriptionally silenced state of genes [8]. Therefore determining of the DNA methylation state of the promoter regions, rather than the level of the corresponding RNAs or proteins, in patient samples promises a better way for both early detection and rationale personalized therapy of the development of chemoresistance of EC cells [9].

For example, the hypermethylation in the promoter regions of APC, RB1, and CDKN2A was found in EC cells [10, 11]. Notably, the PON3 gene was found to be hypermethylated in EC drug-resistant cells and its expression is negatively correlated with EC drug-resistance [12]. However, it remains elusive how these genes regulate or mediate the EC chemoresistance of EC cells.

Multiple C2 domains transmembrane protein 1 (MCTP1) contains two transmembrane regions and three C2 domains of high Ca^2+^-binding affinity [13, 14]. Most C2 domain proteins are either signal transduction enzymes, such as protein kinase C, or membrane trafficking proteins, such as synaptotagmin 1. MCTP1 and MCTP2 have been implicated in various neuropsychiatric diseases [13, 15]. Moreover, previous studies have identified that MCTP1 is associated with the drug resistance in ovarian cancer cell lines [16, 17]. However, it is still unclear whether MCTP1 is involved in the drug resistance of EC cells. The aim of this study was to evaluate whether MCTP1 are involved in this process, using screening methods, we identified that MCTP1 is down-regulated in the EC drug-resistant cells, owing to the hypermethylation at its promoter region. Further functional analysis showed that MCTP1 indeed involves in the EC drug-resistance, the cell migration and apoptosis. All these results might give us hints for the further design of new biomarker for EC clinical therapy.

## RESULTS

### MCTP1 is hypermethylated in drug-resistant esophageal cancer cell lines

**Figure 1:**
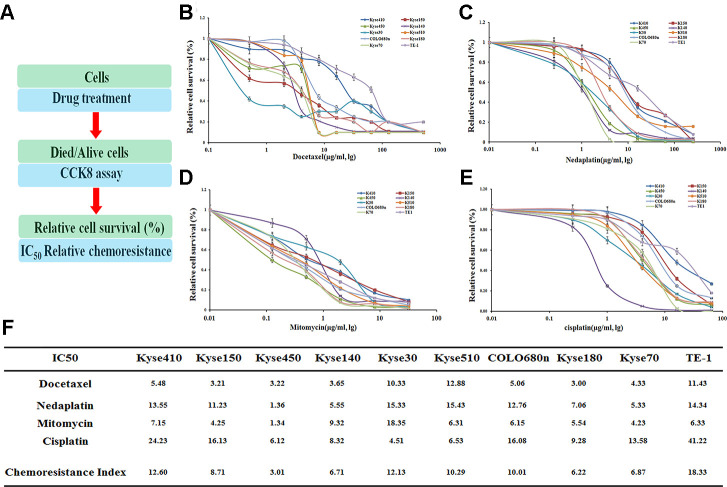
**Drug resistance profiling of ten esophageal cancer cell lines.** (**A**) Experimental scheme. (**B**–**E**) IC_50_ values of the four indicated chemotherapeutics. for ten esophageal cancer cell lines. The cell survival rates were calculated as percentages relative to the mock treatment and plotted against lg μg/ml of drug. (**F**) The IC_50_ values to those of the most sensitive cell cine (Kyse450) are presented in the table.

As found previously, several EC cell lines were identified to be resistant against drugs [12]. The drug dose for 50% cells killed by the following drugs: Docetaxel (Doc), Nedaplatin (Ned), Mitomycin (Mit) and Cisplatin (Cis) after a treatment of 72hr, was determined in the following ten EC cell lines: Kyse410, Kyse150, Kyse450, Kyse140, Kyse30, Kyse510, COLO680n, Kyse180, Kyse70 and TE-1 (Figure 1).

Judged by the chemoresistance index, these cell lines demonstrated varying drug-resistance capabilities against different drugs (Figure 1B–1E). Generally, several cell lines, including TE-1, Kyse410, Kyse30, Kyse510, and COLO680n, have an overall drug resistance index above 10, which show a more significant effect on the drug-resistance than the other five cell lines (Figure 1F). Notably, the TE-1 cell line is most resistant against the above four drugs with the IC_50_ value of 18.33, whereas the Kyse450 is the most drug-sensitive cell line with the IC_50_ value of 3.01.

**Figure 2:**
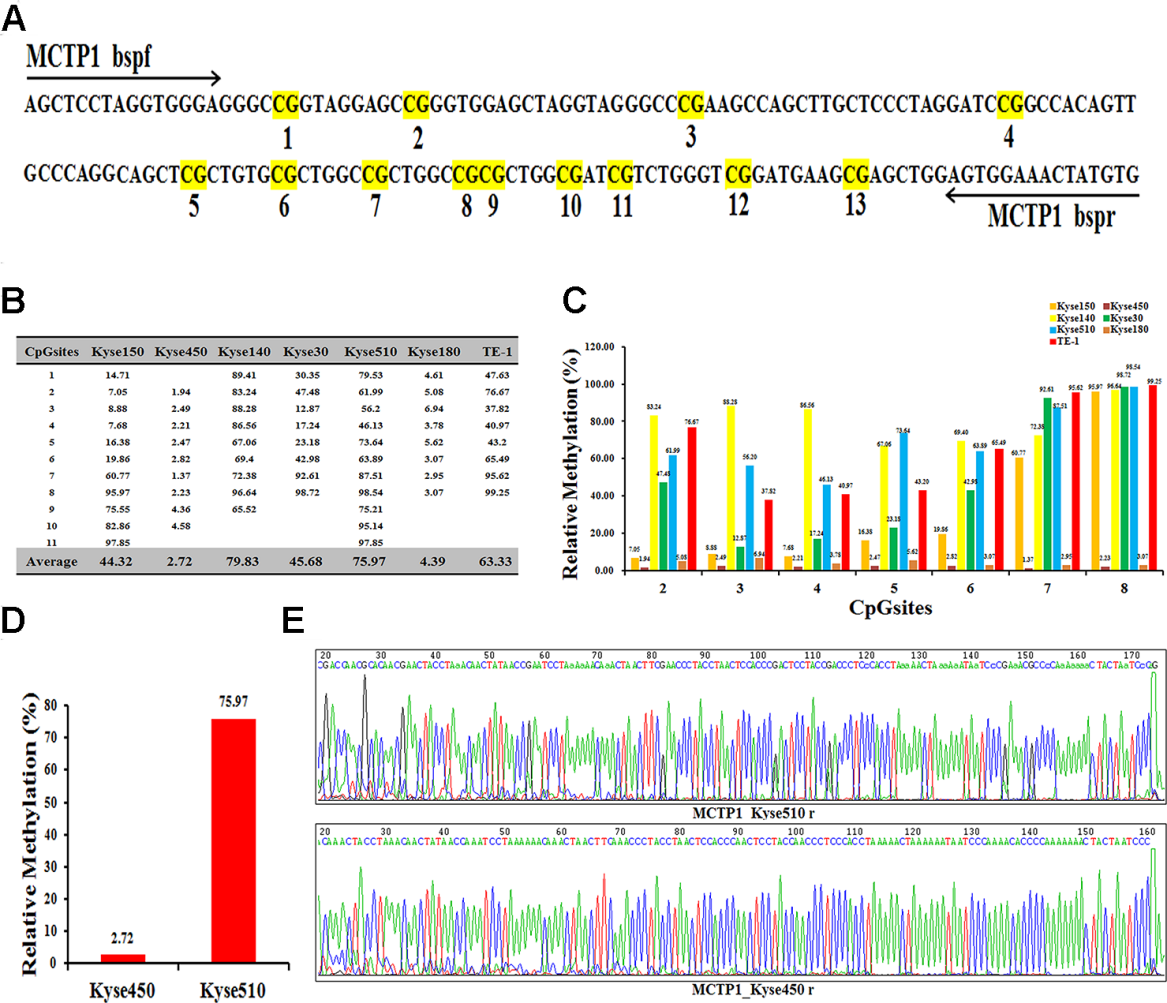
**Differential methylation of the MCTP1 gene in seven esophageal cancer cell lines.** (**A**) BSP primers and CpG dinucleotides of MCTP1 are shown. (**B**) Relative methylation levels (fold) of MCTP1 in seven esophageal cancer cell lines. (**C**) Methylation percentage at seven CpG sites in seven esophageal cancer cell lines. (**D**)The percentage of CpG methylation is summarized in Kyse450 and Kyse510 cells. (**E**) The original sequencing results of the bisulfite-converted DNA are shown in Kyse450 and Kyse510 cells.

To find the insight that affects the drug-resistance of different EC cells, we performed the RNA-seq analysis with the help of UCSC, and found several genes to be differently expressed in EC cells. We selected MCTP1 as our target, which is one of the most differently expressed genes. Notably, the previous studies also indicated that MCTP1 participates in the drug-resistance in ovarian cancer cell lines [16, 18]. Sequence analysis showed that the promoter region of MCTP1 has total 13 CpG sites (Figure 2A). We thus detected the methylation status of the MCTP1 promoter region in seven EC cells by Bisulfite Sequencing PCR (BSP) assay. The results showed that 11 CpG sites among the total 13 CpG sites were methylated at varying ratios (Figure 2B, 2C). Generally, the cell lines Kyse140 and Kyse510 have the highest methylation ratios of 79.83 and 75.97, respectively (Figure 2B). By contrast, the Kyse450 cell line has the lowest methylation ratio of 2.72 (Figure 2B). Generally, the drug-resistance cell lines show a relatively higher methylation ratio, whereas the drug-sensitive cell lines have a much lower methylation ratio. The results clearly indicated that MCTP1 is hypermethylated in the drug-resistant EC cell lines. We selected the drug-resistant Kyse510 cell line with hypermethylation and the drug-sensitive Kyse450 cell line for the further studies (Figure 2D, 2E).

### The expression of MCTP1 is down-regulated in the drug-resistant EC cells

**Figure 3:**
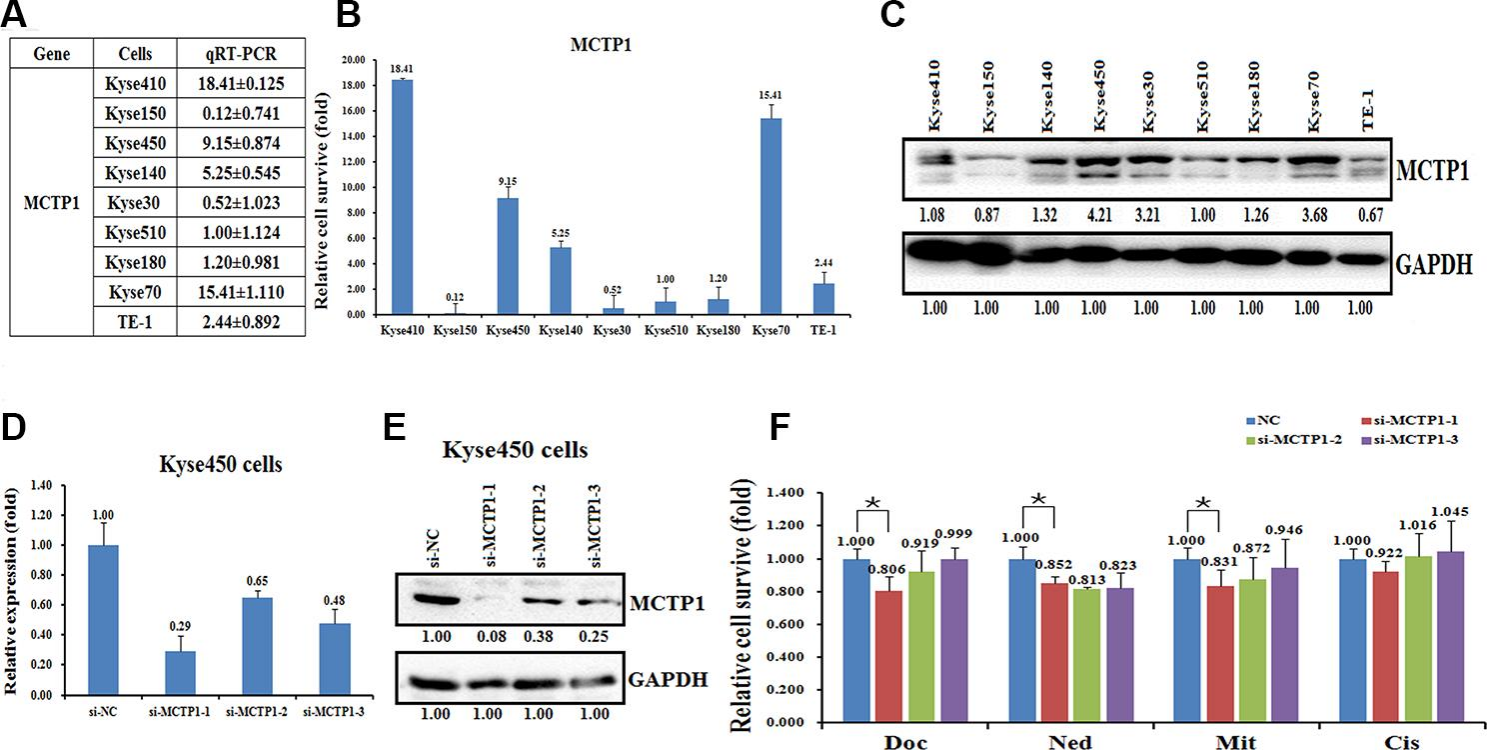
**Effects of a forced reversal of the MCTP1 levels on the drug resistance of Kyse450 cells.** The levels of MCTP1 mRNA (**A** and **B**) and protein level (**C**) determined by qRT-PCR and western blot analysis in the nine esophageal cancer cell lines. The levels of mRNA (**D**) and MCTP1 protein level (**E**) determined by qRT-PCR and western blot analysis in the si-MCTP1-transfected versus the NC-transfected Kyse450 cells. (**F**) The CCK8 assays showing cell death triggered by an IC_50_ dose of drug in Kyse510 cells transfected with three different regions’ si-MCTP1 versus the negative control (NC) assayed 72hr after treatment with the IC_50_ dose of drugs.

To determine whether the hypermethylation may affect the expression of MCTP1 in EC cells, we detected the expression levels of MCTP1 in the nine EC cell lines. The qRT-PCR assay revealed that the transcription of MCTP1 is down-regulated in the drug-resistant cell lines, such as Kyse30, Kyse150, Kyse510, and Kyse180 (Figure 3A, 3B). By contrast, the mRNA levels of MCTP1 are much higher in the drug-sensitive cell lines Kyse450 and Kyse70 (Figure 3A, 3B). In agreement with the expression of MCTP1 mRNA, the expression of MCTP1 proteins are also down-regulated in the drug-resistant cell lines, as shown by the western blot analysis (Figure 3C). The results suggested that MCTP1 expression is down-regulated in the drug-resistant EC cell lines perhaps due to the hypermethylation at the promoter region of MCTP1.

**Figure 4:**
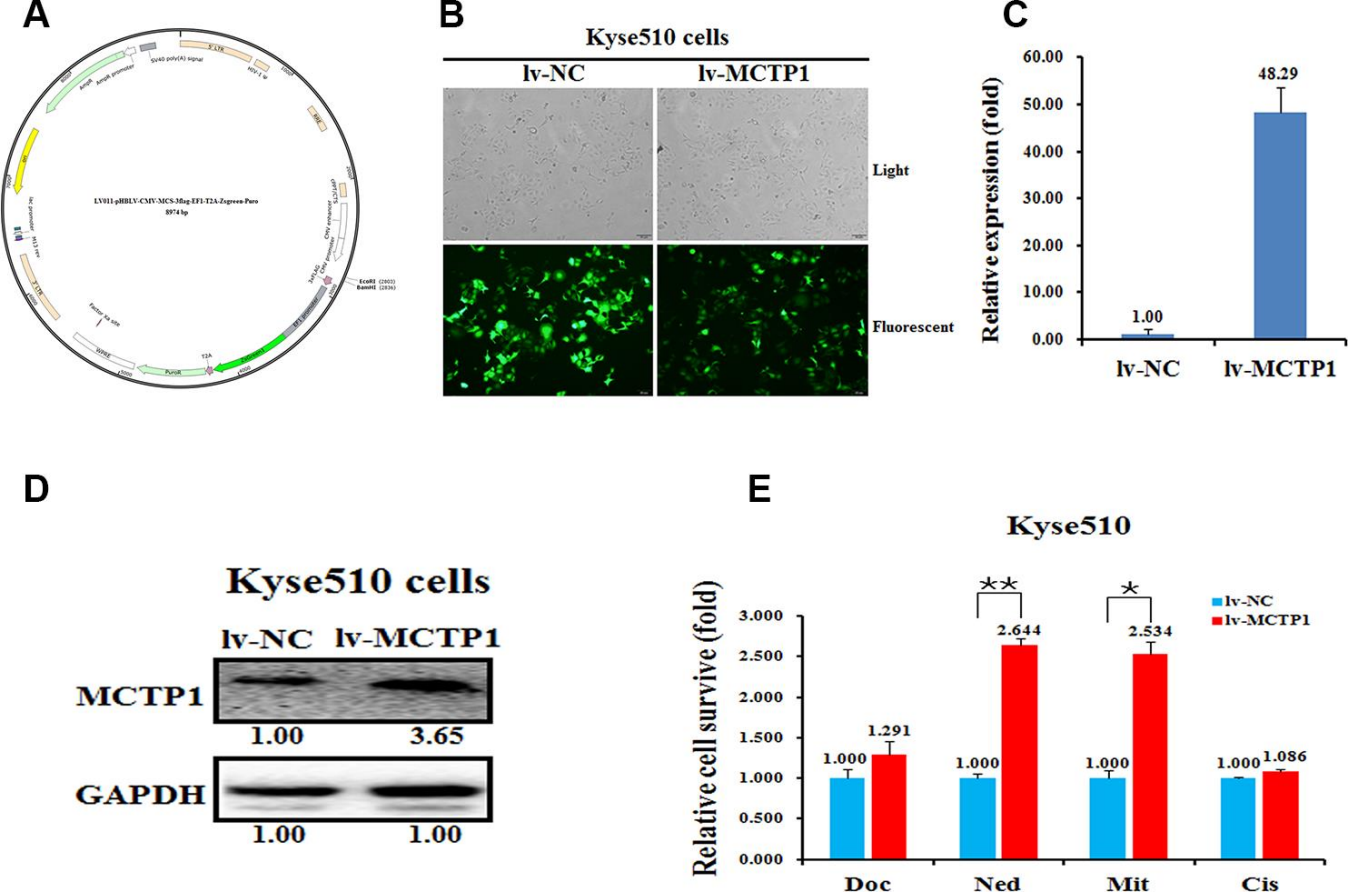
**Effects of a forced reversal of the MCTP1 levels on the drug resistance of Kyse510 cells.** (**A**) MCTP1 overexpression lentivirus vector map. (**B**) Representative areas of light and fluorescent of Kyse510 cells infected with MCTP1 overexpression lentivirus vector and the negative control (NC). MCTP1 mRNA (**C**) and protein (**D**) level determined by qRT-PCR and western blot analysis of Kyse510 cells infected with MCTP1 overexpression lentivirus vector and the negative control (NC). (**E**) The cell death triggered by an IC_50_ dose of four drugs in Kyse510 cells infected with MCTP1 overexpression lentivirus vector versus the negative control (NC) assayed 72hr after treatment with the IC_50_ dose of drugs.

Next, we transfected three si-MCTP1 to down-regulate the MCTP1 level in the drug-sensitive Kyse450 cells and tested the drug-resistance ability against the four drugs. As expected, transfection of three si-MCTP1 significantly down-regulates its expression at both mRNA and protein levels (Figure 3D, 3E). Among the three si-MCTP1, the first si-MCTP1-1 one showed the highest silence ability, and resulted in mRNA and protein levels of only 0.29 and 0.08, respectively, compared to the control (Figure 3D, 3E). Accompanied with the decrease of MCTP1 in Kyse450 cells, the cells are less resistant against the above four drugs, as the relative cell survival ratio is a little bit decreased (Figure 3F). Notably, the si-MCTP1-1 has a most significant role in decreasing the cell survival rate in Kyse450 cells (Figure 3F). Conversely, we over-expressed GFP-tagged MCTP1 in Kyse510 cells to further test the drug-resistance effect (Figure 4A). The fluorescence assays showed that the GFP-MCTP1 construct is indeed expressed in the drug-resistant Kyse510 cells (Figure 4B). As a result, the Kyse510 cells harboring GFP-MCTP1 have a much higher expression of MCTP1, that are 48.29- and 3.65-folds at the mRNA and protein levels, respectively (Figure 4C, 4D). Following the up-regulation of MCTP1 in Kyse510 cells, the cells are somewhat more resistant against the four drugs (Figure 4E). All these results suggest that MCTP1 is positively correlated with the drug-resistance capability of EC cells, despite that the MCTP1 level is much lower in the drug-resistant EC cells.

### MCTP1 affects the migration and apoptosis of EC cells *in vitro*

**Figure 5:**
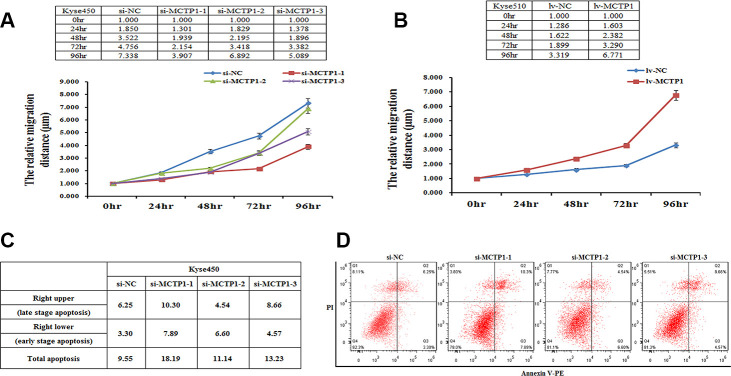
**MCTP1 expression level affecting cell proliferation and apoptosis.** (**A**) The cell proliferation assays showed the lower proliferation capacity of Kyse450 cells when transfected with three different regions’ si-MCTP1 versus the negative control (NC). (**B**) The higher proliferation capacity of Kyse510 cells infected with MCTP1 overexpression lentivirus vector versus the negative control (NC). (**C**, **D**) FACS analysis of the effects of the three different regions’ si-MCTP1 versus the negative control (NC) on apoptosis in Kyse450 cells.

As found previously [19, 20], the hypermethylation of genes may affect the physiological properties of cancer cells. Here we demonstrated that MCTP1 is hypermethylated, which results in the down-regulation of MCTP1 in the drug-resistant EC cells. We then tested whether MCTP1 may affect the migration of EC cells by the wound-healing assays. We first down-regulated MCTP1 by transfection of three si-MCTP1 in Kyse450 cells. Compared to the control cells, along with the time course, transfection of si-MCTP1 significantly increased the migration capability (Figure 5A). By contrast, if we increased the MCTP1 level in Kyse510 cells, the cell migration capability is significantly increased along the time (Figure 5B). The results clearly showed that the MCTP1 level positively correlates with the migration of EC cells.

Then we performed the cell apoptosis assays to check whether MCTP1 may involve in this process. Using the Kyse450 cells with down-regulation of MCTP1 by transfecting si-MCTP1, we clearly found that the apoptosis rate is higher compared to the control cells (Figure 5C, 5D). Notably, the cells at both the late and early apoptosis stages are elevated, with the late-stage apoptosis cells of a majority contribution (Figure 5C, 5D). The results showed that MCTP1 not only confers the drug-resistance of EC cells, but also changes the properties of cell migration and apoptosis.

### The proposed signaling pathway that involves in the MCTP1-mediated drug-resistance of EC cells.

**Figure 6:**
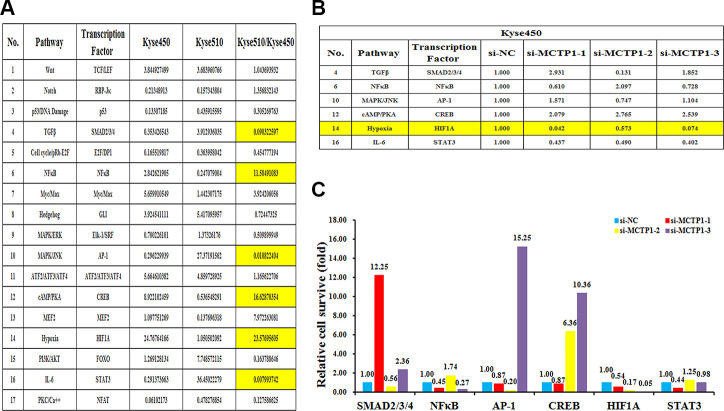
**The effects of the forced reversal of MCTP1 levels on the activity of the signaling pathways in Kyse450 versus Kyse510 cells.** (**A**) The activity of the seventeen pathways in Kyse450 versus Kyse510 cells, and there is great difference among TGFβ, NFκB, MAPK/JNK cAMP/PKA, Hypoxia and IL-6 pathways. (**B**) The relative pathway activities of the three different regions’ si-MCTP1 versus the negative control (NC) transfected in Kyse450 cells. (**C**) The expression ratio of the six transcription Factors in the MCTP1 three different regions siRNA versus the corresponding NC-transfected in Kyse450 cells.

To further elucidate the underlying molecular insights into the MCTP1-mediated EC drug resistance, we measured the transcriptional activities of the seventeen classical signaling pathways in both Kyse510 and Kyse450 cells. The results showed the activities are differentially regulated in Kyse510 versus Kyse450 cells, among which eight pathways showed a higher activity in Kyse510 cells, whereas the other nine pathways had a higher activity in Kyse450 cells. More importantly, totally six pathways TGFβ, NFκB, MAPK/JNK, cAMP/PKA, Hypoxia and IL-6 differed by more than two-folds in Kyse510 and Kyse450 cells, suggesting that they might play a role in EC drug resistance. Among the six pathways, NFκB, cAMP/PKA and Hypoxia are up-regulated in the drug-resistant Kyse510 cells, whereas TGFβ, MAPK/JNK and IL-6 are down-regulated in the Kyse510 cells (Figure 6A). We then compared which of the six pathways correlated with the forced changes of the MCTP1 level in Kyse450 cells. As shown in Figure 6B, 6C, repression of MCTP1 expression of Kyse450 cells by three si-MCTP1 resulted in the up-regulation of cAMP/PKA pathway, but a down-regulation of Hypoxia and IL-6 pathways. As the activity of cAMP/PKA pathway is down-regulated in the Kyse450 cells, which has a higher MCTP1 level. Thus, down-regulation of MCTP1 in Kyse450 cells results in a higher activity of cAMP/PKA pathway, which perfectly meets the negative correlation of cAMP/PKA pathway and the MCTP1 level. Similarly, the changes of the activity of the IL-6 pathway coincides with the positive correlation with the MCTP1 level in the Kyse450 cells. Taken together, we propose that the cAMP/PKA and IL-6 pathways may involve in the MCTP1-mediated drug-resistance of EC cells.

## DISCUSSION

As we know, the epigenetics modifications, such as the altered DNA methylation patterns can influence the expression of genes, and thus affect the cellular functions at every aspect [21]. More importantly, in the past decades, more and more studies have been shown that DNA methylation play roles in drug resistance of cancers [19, 22, 23]. However, it remains elusive how DNA hypermethylation correlates with the drug-resistance of cancers. In our study, we identified that the promoter region of MCTP1 is hypermethylated in drug resistant EC cell lines. The hypermethylation of MCTP1 in return down-regulates its expression in drug resistant EC cells. Furthermore, we showed that the MCTP1 level is positively correlated with the drug-resistance of EC cells. It is controversial with the relatively lower expression level of MCTP1 in drug-resistant cells. It also has an opposite effect with the result from the previous report showing that PON3 is negatively correlated with the drug-resistance of EC cells [12]. The results indicated that MCTP1 and PON3 might apply a totally different mechanism to mediate the EC drug-resistance [24]. One possible explanation of this difference might be the different EC cell lines and drugs used in these two studies. Nevertheless, more investigations are needed to elucidate the fine regulatory networks of these genes in the EC drug-resistance.

The MCTP1 family proteins represent a large member of proteins harboring Ca2+-binding motifs. To date, the members of this family commonly function as either signal transduction enzymes or membrane trafficking proteins [15, 16]. However, only rare cases are reported showing that MCTP1 are related to the cancer development. Here we showed for the first time that MCTP1 indeed participates in the drug-resistance of EC cells. Apparently, MCTP1 is a promising candidate of the biomarker for the drug resistance of EC cells. For the further design of the clinical kit, further studies should be performed to verify the correlation between methylation of MCTP1 and chemotherapy tolerance in esophageal cancer cells, and more clinical samples are needed to verify the reliability of MCTP1 as a marker of chemotherapy tolerance in esophageal cancer.

## CONCLUSIONS

In this work, we identified that MCTP1 participates in the multi-drug resistance of EC cancer, which has implications for the design of new biomarker for the potential therapeutic treatment of EC.

## MATERIALS AND METHODS

### Cell lines and culture

The nine esophageal cancer cell lines: Kyse410, Kyse150, Kyse450, Kyse140, Kyse30, Kyse510, COLO680n, Kyse180, Kyse70 and TE-1 used in this study were bought from the Chinese Academy of Sciences Collection Committee of cultural collections. All cell lines cultured in RPMI 1640 medium (Biological Industries) containing 10% fetal bovine serum (FBS) (PAN Biotech), 1% penicillin/streptomycin (WISENT INC) and 1% glutamine (WISENT INC), and incubated at 37° C in a humidified chamber with 5% CO_2_.

### Chemoresistance profiling (IC_50_ determination)

All of the chemotherapeutic drugs used in this study were of clinical grade. To perform cell proliferation assays, cells in logarithmic growth phase were inoculated into 96-well plate three times at the density of 1.0×10^4^/hole, and treated with the concentration of IC_50_ for 72hr. Then, according to the manufacturer's instructions, cell viability was measured using cell counting kit 8 (CCK-8) (Bimake). The optical density was measured with a 450-nm microplate reader (Tecan). A group that received no drug treatment was used as a reference for calculating the relative cell survival rate [25, 26].

### Bisulfite sequencing PCR (BSP) analysis

Genomic DNA was isolated by DNA Extraction Kit (Thermo Fisher Scientific), verified by electrophoresis on an agarose gel, and treated by an ammonium bisulfite-based bisulfite conversion method. Then the PCR fragments from the converted DNA were sequenced and analyzed. Raw sequence data files were processed, and the area ratio (%) of C over C+T of the primary CpG dinucleotide was calculated as the % of methylation and plotted [19, 27].

### RNA analysis

Total RNA was isolated from the cultured cells with the Trizol (Tiangen, Beijing, China). For mRNA analysis, a cDNA primed by an oligo-dT was constructed using HiScript® RII 1st Strand cDNA Synthesis Kit (Vazyme, Beijing, China). The MCTP1 mRNA level was quantified using duplex-qRT-PCR analysis, wherein TaqMan probes with a different fluorescence profile were used in a FTC-3000P PCR instrument (Funglyn). Using the 2^-ΔΔ^Ct method, target gene expression levels were normalized to the β-actin expression level before the relative levels of the target genes were compared.

### Lentivirus production and infection

Before transfection, 1.3-1.5x10^6^ of the GeneCopoeia HEK293T cells, lentivirus packaging cells or comparable cells were examined and plated in a 10cm dish in 10 ml of DMEM supplemented with 10% heat-inactivated fetal bovine serum so that the cells are 70-80% confluent at the moment of transfection. Incubate the cells at 37° C with 5% CO_2_. 2.5 μg of lentiviral expression plasmid and 5.0 μl (0.5μg/μl) of Lenti-Pac HIV were mixed into 200 μl of Opti-MEM® I (Invitrogen). 15 μl of EndoFectin Lenti was diluted into 200 μl of Opti-MEM I. The diluted EndoFectin Lenti reagent was added dropwise to the DNA solution while gently vortexing the DNA-containing tube. The mixture was then incubated for 10-25 min at room temperature to allow the formation of DNA-EndoFectin complexes. The DNA-EndoFectin Lenti complexes were added directly to each dish, which was gently swirled to distribute the complexes.

### Transient transfection assays and reagents

The siRNA and scrambled (negative control, NC) sequences as well as a riboFECT CP transfection kit were supplied by Guangzhou RiboBio, China. Transfections of the above mentioned ribonucleic acid reagents were performed according to the manufacturer’s instructions [28].

### Western blot protein analysis

Cells were lysed with lysis buffer and heated at 95° C for 10min before electrophoresis/western blot analysis. The primary anti-MCTP1(PA5-42572, Invitrogen) antibodies and anti-GAPDH (60004-1-lg, Proteintech) antibodies were purchased from Proteintech and were recognized with anti-rabbit IgG peroxidase-conjugated antibody (30000-0-AP, Proteintech), followed by an enhanced chemiluminescence reaction (Thermo). Relative levels of proteins were quantified using densitometry with a Gel-Pro Analyzer (Media). The target bands over the GAPDH band were densitometrically quantified, as indicated under each band. All the full-length unprocessed gels of immunoblots were provided in Supplementary Figure 1 of Supplementary materials.

### Cell proliferation assay

The capacity for cellular proliferation was measured by CCK8-based cell proliferation assay. Cells infected lentivirus or control or transfected with MCTP1 siRNA or control siRNA were seeded in 96-well plates at a density of 5x10^3^ cells per well, and cell proliferation assays were performed every 24 hr using CCK8. The number of viable cells was measured by their absorbance at 450nm at the indicated time points.

### Flow cytometry apoptosis analysis

The Kyse450 cells transfected with MCTP1 siRNA or control siRNA were seeded into 6-well plates, harvested after 48hr and rinsed with PBS twice. Cells were treated with 200 μl binding buffer, 5 μl Annexin V-FITC and 5μl propidium iodide (PI). After incubation in the dark for 30min at room temperature, the cell apoptotic rate was measured by flow cytometry (Beckman) and analyzed by Flowjo Software. The experiments were performed independently three times, and a representative is shown.

### Signaling pathway analysis

The cells were transfected in triplicate with each firefly luciferase reporter construct in combination with the Renilla luciferase-based control construct using the riboFECT CP transfection reagent, and both the luciferase activities were measured in the cell extracts 24 hr after transfection. The luciferase activities (luciferase unit) of the pathway reporter relative to those of the negative control in the transfected cells were calculated as a measurement of the pathway activity [29].

### Statistical analysis

The quantitative RT-PCR, cell viability, apoptosis assays and luciferase reporter assays were performed in triplicate, the data are presented as the means, and the error bars indicate the S.D. Excel was used to process the data. The differences were considered statistically significant at p<0.05 using Student’s *t-test*.

## Supplementary Material

Supplementary Figure 1
