# Supplementary material for: The DNA methylation-regulated MCTP1 activates the drug-resistance of esophageal cancer cells
Source: Aging (Albany NY). 2021 Feb 11;13(3):3342–52. doi: 10.18632/aging.104173 (PMC7906193; doi:10.18632/aging.104173)
Supplement: Supplementary Figure 1 [file aging-13-104173-s001.pdf]

SUPPLEMENTARY FIGURE

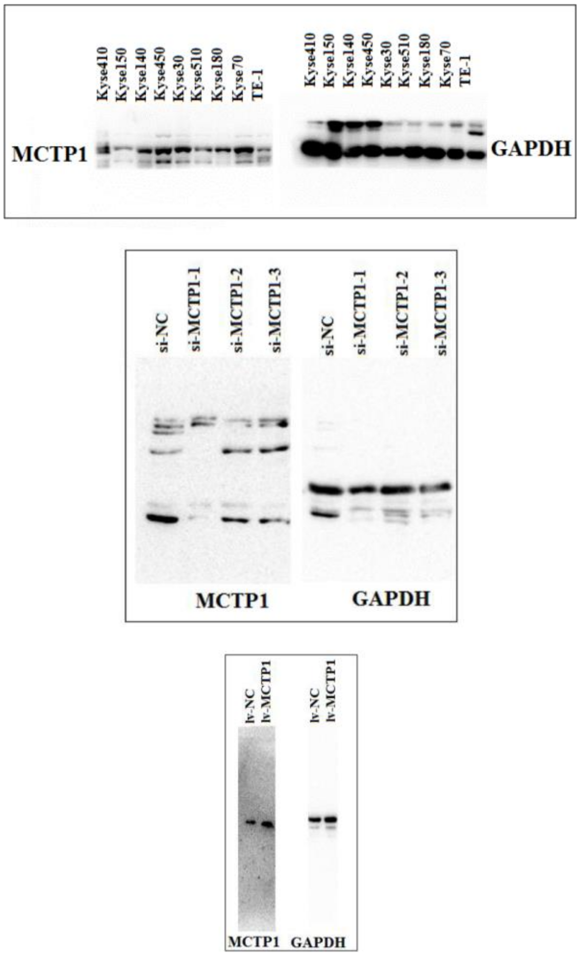

Supplementary Figure 1. The full-length gels of the Figure 3C western analyses used in the revised manuscript. The full-length gels of the Figure 3E western analyses used in the revised manuscript. The full-length gels of the Figure 4D western analyses used in the revised manuscript.
